# Prognosis impact of multiple novel lymphocyte‐based inflammatory indices in patients with initially diagnosed coronary artery disease

**DOI:** 10.1002/iid3.1340

**Published:** 2024-09-27

**Authors:** Yi Gao, Geng Bai, Yuqing Li, Bo Yu, Ziqiang Guo, Xiaolin Chen, Tong Liu, Guangping Li

**Affiliations:** ^1^ Tianjin Key Laboratory of Ionic‐Molecular Function of Cardiovascular Disease, Department of Cardiology, Tianjin Institute of Cardiology The Second Hospital of Tianjin Medical University Tianjin China

**Keywords:** coronary artery disease, major adverse cardiovascular events, novel inflammatory markers, percutaneous coronary intervention

## Abstract

**Background:**

This study aimed to evaluate six novel lymphocyte‐based inflammatory markers (neutrophil‐lymphocyte ratio, monocyte‐lymphocyte ratio, platelet‐lymphocyte ratio [PLR], systemic immune inflammation index [SII], systemic inflammatory response index, and systemic immune inflammation response index [SIIRI]) in patients with newly diagnosed coronary artery disease [CAD].

**Methods:**

A total of 959 patients newly diagnosed with CAD and underwent diagnostic coronary angiography were enrolled in this study and followed for major adverse cardiovascular events (MACEs), including cardiovascular death, nonfatal myocardial infarction, and nonfatal stroke. The best cutoff value was used to compare the six indicators. Cox risk regression analysis evaluated the relationship between novel lymphocyte‐based inflammatory markers and MACEs in newly diagnosed CAD patients.

**Results:**

During a mean follow‐up period of 33.3 ± 9.9 months, 229 (23.9%) MACEs were identified. Multivariate Cox regression analysis showed that only SIIRI (hazard ratio [HR]: 5.853; 95% confidence interval [CI]: 4.092–8.371; *p* < .001) and PLR (HR: 1.725; 95% CI: 1.214–2.452; *p* = .002) were independent predictors of MACEs. Nevertheless, following the adjustment for covariates, only the SIIRI was found to be a significant predictor MACEs and its corresponding specific endpoint occurrences. The predictive ability of the model was improved when six different inflammatory markers were added to the basic model established by traditional risk factors, namely, the C‐index increased, and the SIIRI increased most significantly (AUC: 0.778; 95% CI: 0.743–0.812; *p* < .001). However, among the six novel inflammatory markers, only SIIRI had improved net reclassification improvement (NRI) and integrated discrimination improvement (IDI) (NRI: 0.187; 95% CI: 0.115–0.259, *p* < .001. IDI: 0.135; 95% CI: 0.111–0.159, *p* < .001), which was superior to the basic model established by traditional risk factors.

**Conclusions:**

SIIRI is independent predictor of MACEs in newly diagnosed CAD patients. SIIRI was superior to other measures in predicting MACEs. The combination of SIIRI and traditional risk factors can more accurately predict MACEs.

## Graphical abstract

SIIRI ≥ 247.170 × 10^18^/L^2^ is independently associated with MACEs in newly diagnosed CAD patients. Combining SIIRI with the traditional risk factors model can better predict MACEs.

## 1 INTRODUCTION

Coronary artery disease (CAD) is a progressive disease resulting in restricted cardiac blood flow secondary to atherosclerotic disease in epicardial coronary arteries, characterized by coronary atherosclerotic plaque formation. Thrombosis in the arterial lumen caused by plaque detachment is considered the leading cause of death in patients with CAD.^1^ Although the mortality of CAD has decreased in recent years, it is still the leading cause of death in the United States, resulting in significant social and economic burdens.^2^ The relationship between coronary artery calcification and the development of CAD has been recently well‐studied.^3^ Coronary artery calcification was quantified by coronary angiography. With an increase in the coronary artery calcification score, the incidence of CAD gradually increases.^4^ Patients with higher coronary artery calcification scores have an increased likelihood of having an acute coronary syndrome (ACS).^5^

The effective implementation of primary and secondary prevention strategies, and timely and effective revascularization in patients with chronic CAD can significantly reduce the incidence of major adverse cardiovascular events (MACEs) and mortality, thereby improving the clinical prognosis of patients with CAD.^6^ Despite successful recanalization of the blood vessels by percutaneous coronary intervention (PCI), persistent and recurrent chest pain symptoms have not been totally relieved.^7^ As assessed by the instantaneous wave‐free ratio, residual ischemia remained in about a quarter of patients. Patients without residual ischemia had better relief of angina symptoms at 1 year than those with residual ischemia.^8^ Therefore, accurate and comprehensive risk assessment is particularly important for the clinical diagnosis, treatment, and prognosis of patients with high‐risk CAD after PCI.

Inflammation plays a crucial role in the development of atherosclerosis and is a key mediator and detrimental factor in myocardial ischemia‐reperfusion injury.^9^, ^10^ Inflammatory cells such as white blood cells and new lymphocyte‐based inflammatory markers such as MLR, PLR, and NLR are related to CAD severity and clinical prognoses.^11^, ^12^, ^13^ SII and SIRI, new inflammatory markers combined with three blood cell subtypes, were initially used to predict poor clinical prognosis in cancer patients.^14^, ^15^ In recent years, their ability to predict the clinical prognosis of cardiovascular disease has received much attention. Studies have confirmed they can be used as risk stratification indicators to screen high‐risk populations and are independent predictors of poor clinical prognosis.^16^

The routine use of statins after coronary revascularization is beneficial to the clinical prognosis and disease outcome of patients. The anti‐inflammatory effects of statins help them play an active role in limiting the progression of atherosclerosis.^17^ Antiplatelet drugs can also inhibit the progression of atherosclerosis due to their anti‐inflammatory effects.^18^ Therefore, inflammatory markers may more accurately assess the degree of systemic inflammatory response in patients with newly diagnosed CAD without statins and antiplatelet drugs, and thus predict a poor clinical prognosis.

Recently, systemic immune inflammation response index (SIIRI), an inflammatory marker that combines four blood cell subtypes, has also been shown to be an independent and significant predictor of disease severity in patients with ACS.^19^ However, there is still a lack of studies comparing the ability of different inflammatory markers to predict poor clinical prognosis in patients with newly diagnosed CAD.^20^ Selection of the best predictors, by comparison, will be a good tool for screening patients at high risk of CAD at the initial diagnosis.

Studies have shown that traditional risk factors such as hypertension, diabetes, dyslipidemia, and smoking are closely related to the occurrence and development of CAD.^21^ Therefore, we evaluated the predictive power of lymphocyte‐based inflammatory markers combined with traditional risk factor models.

In this study, we evaluated the ability of neutrophil‐lymphocyte ratio (NLR), platelet‐lymphocyte ratio (PLR), monocyte‐lymphocyte ratio (MLR), systemic immune inflammation index (SII), systemic inflammatory response index (SIRI), and SIIRI to predict poor clinical prognosis in patients with newly diagnosed CAD. We compared the value of different inflammatory markers and traditional risk factors for risk stratification in patients with newly diagnosed CAD.

## 2 MATERIALS AND METHODS

### 2.1 Study population

**Figure 1:**
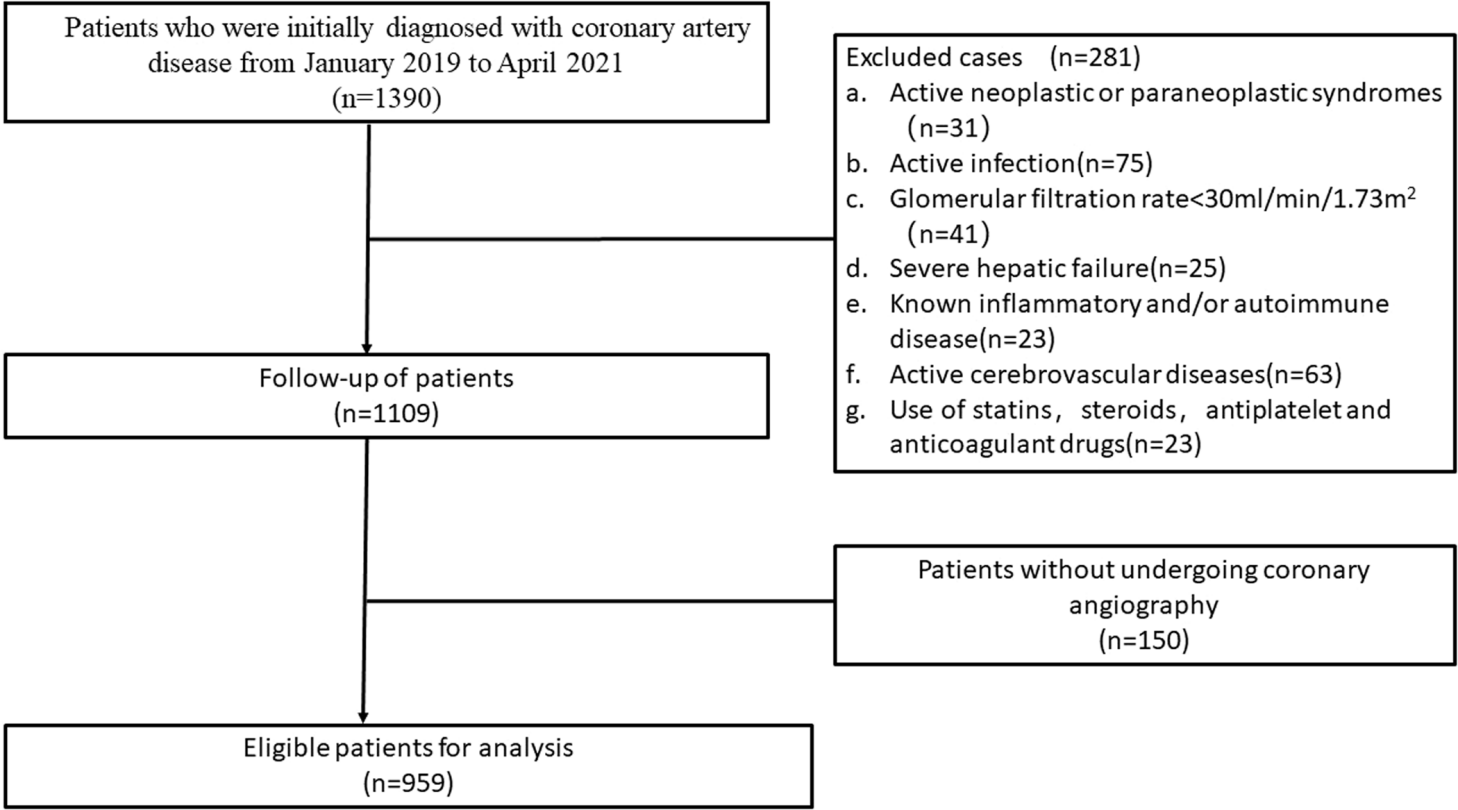
Crowd screening flow chart.

Patients with newly diagnosed CAD who underwent diagnostic coronary angiography in the Second Hospital of XXX Medical University from January 2019 to April 2021 were analyzed in this retrospective study. Each patient signed a written informed consent to participate in the study. Patients who meet the exclusion criteria will be excluded. Exclusion criteria were as follows: (1) active tumor or paraneoplastic syndrome, (2) acute infection, (3) severe renal insufficiency (eGFR <30 mL/min/1.73 m^2^), (4) severe liver failure, (5) known inflammatory/autoimmune disease, (6) active cerebrovascular disease, (7) Use of statins, steroids, antiplatelet and anticoagulant drugs before the onset of illness. As shown in Figure 1. The primary endpoint was MACEs, defined as the composite incidence of cardiovascular death, nonfatal MI, and nonfatal stroke.

All patients had persistent chest pain on hospitalization and the time interval from onset to coronary angiography was less than 6 h. All patients with congestive HF were treated with diuretics before hospitalization. Coronary revascularization (PCI/CABG) or drug conservative therapy was performed depending on the degree of vessel narrowing and in combination with the patient's own wishes.

All patients underwent a complete blood count (CBC) on admission which was included in the statistical analysis.

The independent predictive ability of inflammatory markers was determined by univariate and multivariate Cox regression analysis and verified by the K‐M survival curve. Different inflammatory indicators were added to the basic model established by traditional risk factors to evaluate the diagnostic ability of the model.

### 2.2 Clinical and laboratory data

Electronic medical records collected data on demographic characteristics and laboratory test results. Gaps in medical records were obtained by asking the patient for missing data on admission. Results of the first venous blood sample and CBC were obtained from all hospitalized patients before diagnostic coronary angiography. In the analysis of the biomarkers, NLR is the ratio of neutrophil count to lymphocyte count, PLR is the ratio of platelet count to lymphocyte count, MLR is the ratio of monocyte count to lymphocyte count, SII is defined as platelet count × neutrophil count/lymphocyte count, SIRI as monocyte count × neutrophil count/lymphocyte count, and SIIRI as platelet count × monocyte count × neutrophil count/lymphocyte count. Three independent cardiologists evaluated the coronary angiographic results of each patient. The severity of lesions was quantified using the CASSC score. The degree of stenosis of the main coronary arteries (left anterior descending artery, left circumflex artery, and right coronary artery) >70% was assigned 1 point, and the degree of stenosis of the left main coronary artery >50% was assigned 2 points. The final results on a score of 0–3 were incorporated into the analysis to show the severity of CAD during the onset of CAD in the study patients.

### 2.3 Statistical and analysis

Continuous variables were reported as mean ± standard deviation or median (25th to 75th percentile) and compared using *t*‐tests or Wilcoxon rank‐sum tests when appropriate. Categorical variables are displayed as frequencies and percentages, using Fisher's exact or Chi‐square test, as suitable to determine the significance of categorical variables between the two groups. The receiver operating characteristic (ROC) curve determined the optimal cut‐off value. Kaplan–Meier curves were used for survival analysis to analyze the differences in prognosis and event‐free survival rates of patients in different groups. Primary clinical prognoses were presented as percentages and proportions with 95% confidence intervals (CIs). After adjusting for individual risk factors, univariate and multivariate Cox regression analyzes were used to evaluate the hazard ratios (HRs) for combined and individual endpoints with 95% confidence intervals (CIs). The multivariate analysis included baseline clinical factors that differed significantly between the two groups (*p* < .005). To assess whether adding inflammation markers would improve the ability of a basic model of known risk factors (gender, age, hypertension, diabetes, newly diagnosed hyperlipidemia, smoking history) to predict adverse cardiovascular events, we calculated the C‐index, NRI, and IDI. Two‐tailed *p*‐values <.05 were thought to be statistically significant. All statistical analyzes were performed employing SPSS 27.0, R 4.2.2, and GraphPad Prism 8.0.

## 3 RESULTS

### 3.1 Study design and baseline characteristics

The mean age of the 959 patients at baseline was 61.35 ± 10.79 years, and 51.82% were male. Among the 959 patients, 63.6% had hypertension (*N* = 610), 24.8% had diabetes (*N* = 239), 24.6% had newly diagnosed dyslipidemia (*N* = 236), and 31.9% had a history of smoking (*N* = 306). During a mean follow‐up period of 33.3 ± 9.9 months, MACEs occurred in 229 patients (23.9%). Compared with event‐free patients, MACEs were associated with higher fasting blood glucose levels, glycosylated hemoglobin, triglycerides, and lower albumin and high‐density lipoprotein levels. Patients with MACEs were likelier to have left main CAD, multivessel disease, branch disease, and a higher CASSC score. Before admission, patients with MACEs were less likely to take ACEIs. We adjusted the differences between groups in variables associated with traditional factors. Compared with event‐free patients, patients with MACEs were older, more likely to be male, and more likely to have diabetes, newly diagnosed dyslipidemia, and a smoking history. There was no significant difference in the prevalence of hypertension between the two groups. Table 1 summarizes the baseline characteristics of the study population.

**Table 1:** Basic characteristics of initially diagnosed coronary artery disease.

	ALL (*N* = 959)	No such event (*N* = 730)	MACEs (*N* = 229)	*p*‐value
**Clinical characteristics**				
Age (years)	61.4 (50.6–72.2)	60.6 (50.4–70.8)	63.6 (51.6–75.6)	<.001
Male sex, *n* (%)	497 (51.8%)	358 (49.0%)	139 (60.7%)	.002
Hypertension, *n* (%)	610 (63.6%)	454 (62.2%)	156 (68.1%)	.098
Diabetes mellitus, *n* (%)	238 (24.8%)	157 (21.5%)	81 (35.4%)	<.001
New diagnosis dyslipidemia, *n* (%)	236 (24.6%)	163 (22.3%)	73 (31.9%)	.004
Current smoker, *n* (%)	306 (31.9%)	217 (29.7%)	89 (38.9%)	.009
Congestive heart failure, *n* (%)	63 (6.6%)	46 (6.3%)	17 (7.4%)	.541
Cardiac function classification (Killip)	1.9 (1.3–2.5)	1.9 (1.3–2.5)	1.9 (1.3–2.5)	.247
**Laboratory parameters**				
Hemoglobin (g/L)	140 (125–155)	140 (125–155)	141 (126–156)	.370
White blood cell (10^9^/L)	7.3 (4.8–9.8)	6.9 (4.6–9.2)	8.8 (5.9–11.7)	<.001
Neutrophil (10^9^/L)	4.9 (2.7–7.1)	4.5 (2.6–6.4)	6.4 (3.8–9.0)	<.001
Monocyte (10^9^/L)	0.4 (0.2–0.6)	0.4 (0.2–0.6)	0.5 (0.3–0.7)	<.001
Lymphocyte (10^9^/L)	1.8 (1.1–2.5)	1.8 (1.1–2.5)	1.8 (0.9–2.7)	.162
Platelet (10^9^/L)	226 (169–283)	224 (168–280)	234 (172–296)	.022
Glycosylated hemoglobin (%)	5.4 (4.0–6.8)	5.3 (4.0–6.6)	5.6 (4.3–6.9)	.001
Fasting blood glucose (mmol/L)	6.3 (4.4–8.2)	6.2 (4.4–8.0)	6.7 (4.4–9.0)	<.001
Total cholesterol (mmol/L)	5.0 (3.9–6.1)	5.0 (3.9–6.1)	5.0 (3.9–6.1)	.942
Triglycerides (mmol/L)	1.7 (0.7–2.7)	1.7 (0.8–2.6)	1.8 (0.8–2.8)	.044
High‐density lipoprotein (mmol/L)	1.9 (0.9–2.9)	1.2 (0.9–1.5)	1.1 (0.8–1.4)	.003
Low‐density lipoprotein (mmol/L)	3.2 (2.3–4.1)	3.2 (2.3–4.1)	3.2 (2.3–4.1)	.524
Urea nitrogen (mmol/L)	6.0 (2.1–9.9)	5.9 (1.9–9.9)	6.0 (2.8–9.2)	.823
Creatinine (umol/L)	68.3 (47.0–89.6)	67.9 (46.4–89.4)	69.3 (49.0–89.6)	.386
Uric acid (umol/L)	335.2 (242.9–427.5)	332.7 (240.0–425.4)	343.3 (252.6–434.0)	.128
Albumin (g/L)	42.1 (38.6–45.6)	42.5 (39.2–45.8)	40.8 (37.1–44.5)	<.001
**Coronary artery disease**				
Left main coronary artery disease, *n* (%)	44 (4.6%)	23 (3.2%)	21 (9.2%)	<.001
Polyvascular disease, *n* (%)	428 (44.6%)	272 (37.3%)	156 (68.1%)	<.001
Branch lesions, *n* (%)	258 (26.9%)	177 (24.3%)	81 (35.4%)	<.001
CASSC score	1.3 (0.2–2.4)	1.1 (0.1–2.1)	1.8 (1.0–2.6)	<.001
**Medications**				
ACEI, *n* (%)	27 (2.8%)	25 (3.4%)	2 (0.9%)	.040
ARB, *n* (%)	221 (23.0%)	168 (23.0%)	53 (23.1%)	.928
β‐biockers, *n* (%)	117 (12.2%)	86 (11.8%)	31 (13.5%)	.487
CCB, *n* (%)	293 (30.6%)	212 (29.0%)	81 (35.4%)	.070
Diuretics, *n* (%)	63 (6.6%)	46 (6.3%)	17 (7.4%)	.541
Glucose‐lowering drugs, *n* (%)	222 (23.2%)	167 (22.9%)	55 (24.0%)	.654
**Lymphocyte‐based inflammatory indices**				
Neutrophil‐lymphocyte ratio (NLR)	3.2 (0.9–5.5)	2.8 (0.9–4.7)	4.4 (1.6–7.2)	<.001
Platelet‐lymphocyte ratio (PLR)	139.6 (80.0–199.2)	135.5 (77.3–193.7)	152.8 (90.7–214.9)	<.001
Monocyte‐lymphocyte ratio (MLR)	0.3 (0.1–0.5)	0.2 (0.1–0.3)	0.3 (0.2–0.4)	<.001
Systemic inflammatory index (SII) [10^9^/L]	706.6 (174.0–1239.2)	621.0 (148.0–1094.0)	980.7 (365.7–1595.7)	<.001
Systemic inflammatory response index (SIRI) [10^9^/L]	1.4 (0.1–2.7)	1.1 (0.1–2.1)	2.2 (0.2–4.2)	<.001
Systemic immune‐inflammation response index (SIIRI) [10^18^/L^2^]	319.7 (0.00–639.4)	259.4 (7.0–511.8)	512.8 (4.9–1020.7)	<.001

Abbreviations: ACEI, angiotensin‐converting enzyme inhibitors; ARB, angiotensin receptor blockers; CCB, calcium channel blockers; N, number.

### 3.2 Comparison of different novel lymphocyte‐based inflammatory markers

A comparison of the optimal cutoff values and the prediction of MACEs for other novel lymphocyte‐based inflammatory markers is shown in Table 2. By drawing the ROC curve, the optimal cutoff value was obtained according to the Youden index. We observed that SIIRI had the highest sensitivity (82.0%) and NLR had the highest specificity (73.6%). The area under the AUC curve of different lymphocyte‐based inflammatory markers was 0.753 [95% CI: 0.717–0.788] for SIIRI, 0.753 [95% CI: 0.717–0.789] for SIRI, and 0.738 [95% CI: 0.738] for SII, and 0.694 [95% CI: 0.654–0.733] for MLR, and 0.592 [95% CI: 0.548–0.635] for PLR, and 0.725 [95% CI: 0.686–0.763] for NLR. SIIRI and SIRI appeared to perform best based on the area under the AUC curve.

**Table 2:** Different inflammation markers’ cutoff, values, AUC curve area, sensitivity, and specificity.

AAEs	Cut‐off value	AUC (95% CI)	*p*‐value	sensitivity	specificity
SIIRI	247.170 × 10^18^/L^2^	0.753 (0.717–0.788)	**<.001**	0.820	0.609
SIRI	1.199 × 10^9^/L	0.753 (0.717–0.789)	**<.001**	0.754	0.657
SII	681.267 × 10^9^/L	0.738 (0.700–0.775)	**<.001**	0.689	0.715
MLR	0.216	0.694 (0.654–0.733)	**<.001**	0.759	0.544
PLR	136.882	0.592 (0.548–0.635)	**<.001**	0.566	0.606
NLR	3.119	0.725 (0.686–0.763)	**<.001**	0.632	0.736

*Note*: Bold values are statistically significant.

Abbreviations: AUC, the area under the receiver operating characteristic curve; CI, confidence interval; MLR, monocyte‐to‐lymphocyte ratio; NLR, neutrophil‐to‐lymphocyte ratio; PLR, platelet‐to‐lymphocyte ratio; SII, systemic inflammation index; SIRI, systemic inflammation response index; SIIRI, systemic immune‐inflammation response index.

### 3.3 Novel lymphocyte‐based inflammatory markers as independent predictors of MACEs

**Figure 2:**
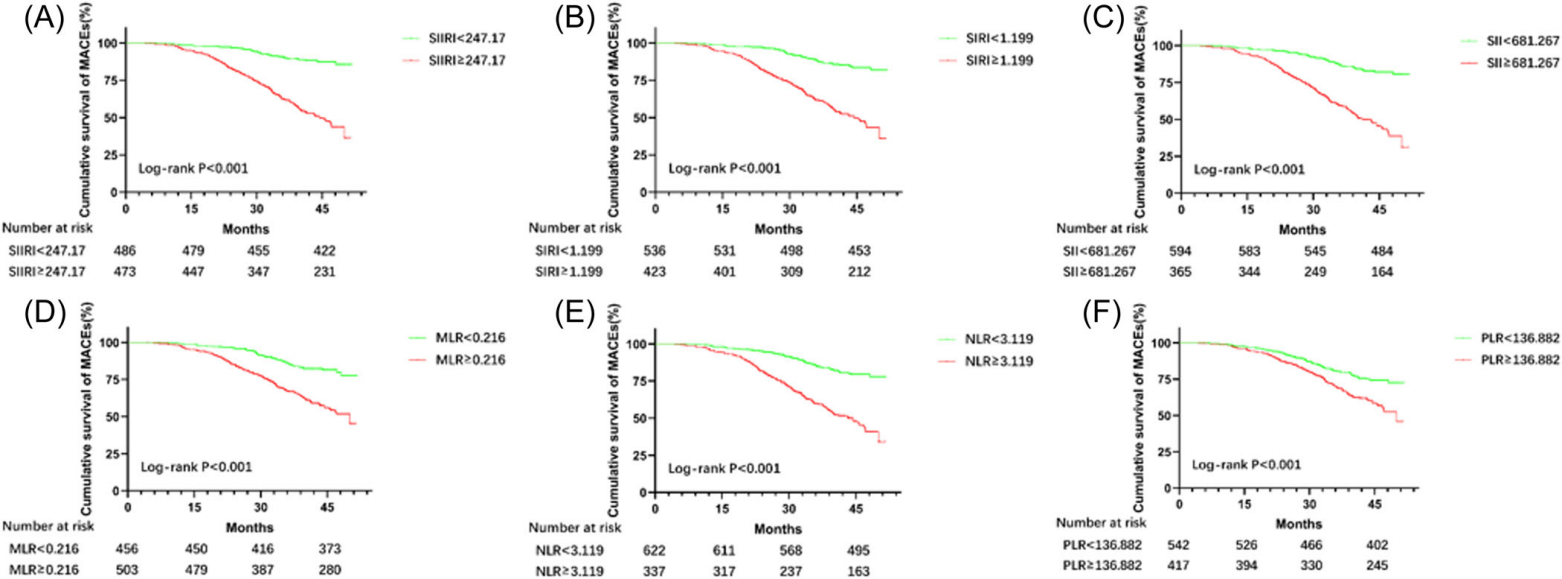
The K‐M survival curves for inflammatory markers. MACEs include cardiac death, nonfatal myocardial infarction, and nonfatal stroke. (A) K‐M survival curves for SIIRI; (B) K‐M survival curves for SIRI; (C) K‐M survival curves for SII; (D) K‐M survival curves for MLR; (E) K‐M survival curves for NLR; (F) K‐M survival curves for PLR. MACE, major adverse cardiovascular event; MLR, monocyte‐to‐lymphocyte ratio; NLR, neutrophil‐to‐lymphocyte ratio; PLR, platelet‐to‐lymphocyte ratio; SII, systemic inflammation index; SIIRI, systemic immune‐inflammation response index; SIRI, systemic inflammation response index.

Table 3 summarizes the results of univariate and multivariate Cox proportional hazards regression analyzes of novel lymphocyte‐based inflammatory markers for predicting the occurrence of MACEs. Factors with *p* < .05 in all univariate analyzes were included in subsequent multivariate analyzes. Univariate Cox proportional hazards regression analysis showed that a higher incidence of MACEs was associated with a higher incidence of SIIRI [HR: 5.162; 95% CI: 3.680–7.241; *p* < .001], SIRI [HR: 1.000; 95% CI: 1.000–1.001; *p* < .001], SII [HR: 1.211; 95% CI: 1.168–1.255; *p* < .001], MLR [HR: 12.811; 95% CI: 7.293–22.505; *p* < .001), NLR (HR: 1.154; 95% CI: 1.115–1.193; *p* < .001] and PLR (HR: 1.003; 95% CI: 1.001–1.005; *p* < .001]. All statistically significant univariate variables with a *p* < .005 were included for further multivariate analysis. In the multivariate Cox proportional hazards regression analysis, only SIIRI [HR: 5.853; 95% CI: 4.092–8.371; *p* < .001] and PLR [HR: 1.725; 95% CI: 1.214–2.452; *p* = .002] remained significantly associated with MACEs. As shown in Figure 2, Kaplan–Meier survival curves showed that patients with higher levels of the novel lymphocyte‐based inflammation index had a higher risk of MACEs (*p* < .001 for all log‐rank).

**Table 3:** The univariable and multivariable Cox regression analysis.

	Univariable Cox regression		Multivariable Cox regression	
	HR (95% CI)	*p*‐value	HR (95% CI)	*p*‐value
Age	1.023 (1.010–1.036)	**<.001**	0.987 (0.973–1.001)	.063
Gender	0.715 (0.548–0.933)	**.013**	1.200 (0.862–1.671)	.280
Hypertension	1.329 (1.005–1.758)	**.046**	0.933 (0.701–1.242)	.634
Diabetes mellitus	1.869 (1.425–2.452)	**<.001**	1.491 (0.946–2.349)	.086
New diagnosis dyslipidemia	1.577 (1.193–2.083)	**.001**	0.830 (0.598–1.151)	.264
Current smoker	1.351 (1.035–1.763)	**.027**	1.027 (0.748–1.409)	.869
Total cholesterol	0.996 (0.891–1.115)	.950		
Triglycerides	1.161 (1.029–1.308)	**.015**	0.912 (0.779–1.067)	.248
High‐density lipoprotein	0.500 (0.307–0.813)	**.005**	0.915 (0.588–1.424)	.693
Low‐density lipoprotein	1.030 (0.892–1.190)	.684		
Urea nitrogen	1.005 (0.977–1.034)	.720		
Creatinine	1.002 (0.998–1.007)	.313		
Uric acid	1.001 (0.999–1.002)	.283		
Glycosylated hemoglobin	1.128 (1.047–1.215)	**.001**	0.909 (0.781–1.058)	.218
Fasting blood glucose	1.105 (1.049–1.164)	**<.001**	0.991 (0.907–1.082)	.836
Albumin	0.899 (0.866–0.933)	**<.001**	0.958 (0.917–1.000)	.052
Left main coronary artery disease	2.231 (1.424–3.496)	**<.001**	0.530 (0.213–1.320)	.173
Poly‐vascular disease	2.971 (2.246–3.931)	**<.001**	1.198 (0.771–1.863)	.422
Branch lesions	1.497 (1.141–1.963)	**.004**	1.022 (0.732–1.428)	.897
CASSC score	1.712 (1.515–1.935)	**<.001**	1.006 (0.810–1.249)	.960
SIIRI ≥ 247.170 × 10^18^/L^2^	5.162 (3.680–7.241)	**<.001**	5.853 (4.092–8.371)	**<.001**
SIRI ≥ 1.199 × 10^9^/L	1.000 (1.000–1.001)	**<.001**	1.164 (0.788–1.719)	.445
SII ≥ 681,267 × 10^9^/L	1.211 (1.168–1.255)	**<.001**	0.857 (0.552–1.332)	.494
MLR ≥ 0.216	12.811 (7.293–22.505)	**<.001**	0.811 (0.592–1.111)	.192
NLR ≥ 3.119	1.154 (1.115–1.193)	**<.001**	0.954 (0.621–1.467)	.830
PLR ≥ 136.882	1.003 (1.001–1.005)	**<.001**	1.725 (1.214–2.452)	**.002**
ACEI	0.376 (0.093–1.512)	.168		
ARB	0.986 (0.725–1.341)	.929		
β‐biockers	1.030 (0.705–1.505)	.877		
CCB	1.258 (0.959–1.650)	.097		
Diuretics	1.250 (0.762–2.050)	.376		
Glucose‐lowering drugs	1.044 (0.770–1.414)	.783		

*Note*: Bold values are statistically significant.

Abbreviations: ACEI, angiotensin‐converting enzyme inhibitors; ARB, angiotensin receptor blockers; CCB, calcium channel blockers; CI, confidence interval; MLR, monocyte‐to‐lymphocyte ratio; NLR, neutrophil‐to‐lymphocyte ratio; PLR, platelet‐to‐lymphocyte ratio; SII, systemic inflammation index; SIRI, systemic inflammation response index; SIIRI, systemic immune‐inflammation response index.

### 3.4 The predictive ability of different novel lymphocyte‐based inflammatory markers

The predictive effects of different novel inflammatory markers on specific endpoints are shown in Table 4. After adjusting for covariates, with the exception of cardiac death, SIIRI demonstrates a strong predictive capability for MACE [HR: 3.830, 95% CI: (2.674–5.587), *p* < .001], nonfatal MI [HR: 4.689, 95% CI: (2.881–7.631), *p* < .001], nonfatal stroke [HR: 4.063, 95% CI: (2.008–8.222), *p* < .001], rehospitalization for congestive HF [HR: 4.559, 95% CI: (2.819–7.373), *p* < .001], and revascularization [HR: 2.470, 95% CI: (1.732–3.521), *p* < .001]. Conversely, other inflammatory markers do not exhibit significant predictive value.

**Table 4:** The association of inflammatory markers and future adverse events in patients.

	NLR	MLR	PLR	SII	SIRI	SIIRI
MACEs	0.864 (0.641–1.166) *p* = .340	0.818 (0.628–1.067) *p* = .138	0.884 (0.671–1.165) *p* = .381	0.773 (0.574–1.041) *p* = .090	0.952 (0.712–1.274) *p* = .742	3.830 (2.674–5.487） ***p*** < **.001**
Cardiac death	1.061 (0.448–2.510) *p* = .893	0.640 (0.291–1.408) *p* = .267	2.175 (0.978–4.839) *p* = .057	1.021 (0.439–2.375) *p* = .961	1.481 (0.637–3.443) *p* = .362	1.389 (0.596–3.236）*p* = .446
Nonfatal myocardial infarction	0.774 (0.522–1.146) *p* = .200	0.845 (0.599–1.193) *p* = .339	0.837 (0.584–1.199) *p* = .332	0.698 (0.472–1.032) *p* = .071	0.758 (0.517–1.112) *p* = .157	4.689 (2.881–7.631） ***p*** < **.001**
Nonfatal stroke	1.019 (0.591–1.755) *p* = .947	0.804 (0.493–1.310) *p* = .381	0.685 (0.405–1.160) *p* = .159	0.873 (0.508–1.501) *p* = .624	1.279 (0.756–2.163) *p* = .360	4.063 (2.008–8.222） ***p*** < **.001**
Hospitalization for congestive heart failure	1.032 (0.696–1.531) *p* = .874	0.686 (0.456–1.013) *p* = .054	0.697 (0.460–1.123) *p* = .071	0.881 (0.591–1.315) *p* = .536	0.997 (0.676–1.470) *p* = .989	4.559 (2.819–7.373） ***p*** < **.001**
Revascularization (PCI/CABG)	0.888 (0.637–1.238) *p* = .483	0.927 (0.689–1.246) *p* = .615	1.138 (0.841–1.541) *p* = .403	0.793 (0.570–1.105) *p* = .170	0.896 (0.646–1.243) *p* = .510	2.470 (1.732–3.521） ***p*** < **.001**

*Note*: MACEs include cardiac death, nonfatal myocardial infarction, and nonfatal stroke. Variable control: adjusted with age, gender, smoking, history of hypertension, diabetes, a new diagnosis of dyslipidemia, albumin, left main coronary artery disease, poly‐vascular disease, branch lesions, and CASSC score. Bold values are statistically significant.

Abbreviations: MACE, major adverse cardiovascular event; MLR, monocyte‐to‐lymphocyte ratio; NLR, neutrophil‐to‐lymphocyte ratio; NRI, net reclassification improvement; PLR, platelet‐to lymphocyte ratio; SII, systemic inflammation index; SIIRI, systemic immune‐inflammation response index; SIRI, systemic inflammation response index.

### 3.5 Combining novel lymphocyte‐based inflammation indices with traditional risk factor models

To assess whether adding novel lymphocyte‐based markers of inflammation to traditional risk factors would improve predictive power, we developed seven models that included traditional risk factors. Compared with the basic model composed of traditional risk factors, the model combining new inflammatory markers with traditional risk factors had a better discrimination ability for MACEs. We observed that there was no significant difference between SIIRI [C‐index: 0.778; 95% CI: 0.743–0.812, *p* < .001], SIRI [C‐index: 0.761; 95% CI: 0.724–0.798, *p* < .001], SII [C‐index: 0.752; 95% CI: 0.715–0.788, *p* < .001], MLR [C‐index: 0.723; 95% CI: 0.684–0.762, *p* < .001], NLR [C‐index: 0.778; 95% CI: 0.705–0.780, *p* < .001], PLR [C‐index: 0.682; 95% CI: 0.642–0.722, *p* < .001], C‐statistics increased significantly. Among the six new models, only the SIIRI combined with traditional risk factors showed greatly improved reclassification performance, with an NRI of 18.7% (*p* < .001) and IDI of 13.5% (*p* < .001). As shown in Table 5.

**Table 5:** Evaluation of predictive models for MACEs.

Model	C‐index (95% CI)	*p*‐value	NRI (95% CI)	*p*‐value	IDI (95% CI)	*p*‐value
Traditional risk factors	0.665 (0.625–0.705)			*p *= ref		*p* = ref
Traditional + SIIRI	0.778 (0.743–0.812)	***p*** < **.001**	0.187 (0.115–0.259)	***p*** < **.001**	0.135 (0.111–0.159)	*p* < .001
Traditional + SIRI	0.761 (0.724–0.798)	***p*** < **.001**	0.079 (−0.096–0.253)	*p* = .377	0.001 (−0.001–0.001)	*p* = .727
Traditional + SII	0.752 (0.715–0.788)	***p*** < **.001**	0.045 (−0.019–0.109)	*p* = .167	0.002 (−0.002–0.006)	*p* = .246
Traditional + MLR	0.723 (0.684–0.762)	***p*** < **.001**	0.017 (−0.032–0.065)	*p* = .506	0.002 (−0.001–0.006)	*p* = .198
Traditional + NLR	0.742 (0.705–0.780)	***p*** < **.001**	0.002 (−0.002–0.006)	*p* = .317	0.001 (−0.001–0.002)	*p* = .652
Traditional + PLR	0.682 (0.642–0.722)	***p*** < **.001**	0.006 (−0.006–0.018)	*p* = .316	0.002 (−0.001–0.001)	*p* = .827

*Note*: MACEs include cardiac death, nonfatal myocardial infarction, and nonfatal stroke. Traditional cardiovascular risk factors model: age, gender, hypertension, diabetes mellitus, new diagnosis dyslipidemia, and current smoker. Bold values are statistically significant.

Abbreviations: AUC, the area under the receiver operating characteristic curve; CI, confidence interval; IDI, integrated discrimination improvement; MACE, major adverse cardiovascular event; MLR, monocyte‐to‐lymphocyte ratio; NLR, neutrophil‐to‐lymphocyte ratio; NRI, net reclassification improvement; PLR, platelet‐to lymphocyte ratio; SII, systemic inflammation index; SIIRI, systemic immune‐inflammation response index; SIRI, systemic inflammation response index.

## 4 DISCUSSION

In this observational study, we evaluated the predictive value of six novel lymphocyte‐based inflammatory markers in predicting MACEs in patients with newly diagnosed CAD. Among these six novel inflammatory markers based on blood cell analysis obtained from patients' venous blood drawn at admission, only SIIRI ≥ 247.170 × 10^18^/L^2^ is independently associated with MACEs in newly diagnosed CAD patients. Otherwise, when combined with traditional risk factors, only the NRI and IDI of SIIRI increased, although the C‐index of all six indicators increased significantly. SIIRI is independent risk factor for MACEs in patients with newly diagnosed CAD. SIIRI was superior to the other five indicators in predicting MACEs. The combination of SIIRI and traditional risk factors can more accurately predict MACEs.

Since Anderson et al.^22^ used the CBC risk score to assess the mortality of patients with cardiovascular disease in 2007, CBC has attracted wide attention due to its simplicity, economy and easy access. Among these parameters, the red cell distribution width (RDW) appears to be a promising predictor. Patel et al.^23^ found that RDW could be an effective predictor of mortality among community‐dwelling older adults with or without age‐related diseases, but the mechanism is unclear. Hunziker et al.^24^ demonstrated that RDW can significantly improves risk stratification of the simplified acute physiology score in intensive care unit (ICU) patients and thereby predicting short‐ and long‐term mortality in ICU patients. During the COVID‐19 epidemic, Arbel et al.^25^ found that RDW was associated with an increased risk of death among patients infected with COVID‐19. The relationship between inflammatory cells such as white blood cells and lymphocytes and the degree of systemic inflammatory response of patients has been confirmed, and the predictive value of novel inflammatory markers has been continuously explored. Inflammatory markers have become an effective predictor for screening high‐risk patients and evaluating their clinical prognosis.

Many studies have shown that neutrophils promote the development of atherosclerosis. Arble et al.^26^ found that NLR was independently associated with the severity of lesions in patients with CAD, and that patients with an NLR > 3 had a worse clinical prognosis. NLR appears to result from the additive effect of conventional risk factors and biomarkers.^26^ Leukocytosis, especially neutrophilia, is a factor in the development of atherosclerosis.^27^ Lymphocytes are involved in immune regulation, and lymphopenia positively correlates with MACEs, HF, and adverse cardiovascular events in patients with ACS.^28^, ^29^ A meta‐analysis of observational studies on NLR has shown that NLR predicts all‐cause mortality and cardiovascular events in patients undergoing revascularization.^30^ However, this study also pointed out that the experimental design focused on observational studies, and the application of NLR in clinical practice needs to be proved by additional trials. Of note, Verdoia et al.^31^ found that patients with a high NLR had a reduced platelet inhibitory capacity even with dual antiplatelet therapy, which increased the risk of thrombosis and ischemic events.

Increased platelets rapidly accelerate the progression of atherosclerosis, predispose to the rupture of vulnerable plaques, and are associated with an increased incidence of adverse cardiovascular events.^32^ In contrast, platelets can regulate the recruitment of inflammatory cells to atherosclerotic plaques and play an immunomodulatory role.^33^ Murat et al. showed that PLR was positively correlated with the Gensini score which indicates the severity of atherosclerotic lesions. PLR could be used as a stratification index for the severity of atherosclerosis.^34^ San et al.^35^ also showed that PLR can be used as an independent predictor of coronary severity in patients undergoing elective coronary angiography. The result in our study is consistent with their findings. PLR can be used as an independent predictor to predict MACEs in patients with newly diagnosed CA, but its predictive power is weak, and the predictive value is low. Therefore, we do not recommend using PLR alone for predicting adverse cardiovascular events. It is worth noting that a cross‐sectional study demonstrated that PLR values are positively correlated with the Gensini score, Syntax score, and coronary artery calcification score.^36^ PLR, combined with other indicators, may synergistically predict the poor clinical prognosis of patients with CAD. In the acute phase of coronary atherosclerotic heart disease, the use of appropriate doses of antiplatelet therapy may help reduce the probability of adverse events, but the associated risk of bleeding is increased.^37^

The migration and maturation of monocytes into macrophages in the arterial wall is the initial event of atherosclerosis.^38^ Macrophages transform into foam cells by the uptake of oxidized lipoproteins, activate various inflammatory factors and oxidative free radicals around atherosclerotic plaques, and promote the process of atherosclerosis.^39^, ^40^ Ji et al.^41^ showed that MLR was positively correlated with Syntax score and was an independent predictor of the severity of CAD.

SII and SIRI were initially used to evaluate the poor clinical prognosis of cancer patients.^14^ The application of SII and SIRI in the diagnosis of CAD and clinical prognosis has been widely discussed in recent years. Compared with NLR, MLR, and PLR, SII and SIRI are composed of three different subtypes of inflammatory cells, but whether their predictive ability can be improved remains to be proved. After excluding related risk factors, SII and SIRI were independent predictors of MACEs within 1 year.^19^ SII can significantly improve the risk stratification of MACEs in patients with CAD, and its predictive effect on MACEs is better than traditional risk factors.^42^ Urbanowicz et al.^43^ found that SIRI may help to diagnose CAD in angina‐equivalent patients, and patients with SIRI ≥ 1.22 are more likely to have single or complex CAD.^44^ A recent meta‐analysis has also shown that SII may be a potential biomarker for the occurrence of cardiovascular disease, and increased SII can increase the risk of cardiovascular disease. However, the level of evidence is generally low, and the optimal cutoff value and suitable population have yet to be determined.^43^

SIIRI is a combination of four different inflammatory cell subtypes and has recently been used to assess the severity of lesions in patients with ACS.^19^ By integrating the changes of platelets, white blood cells, monocytes and lymphocytes in patients with CAD, SIIRI may help to more accurately reflect the systemic inflammatory response. However, whether SIIRI has a stronger predictive value than other inflammatory markers remain to be investigated. Similarly, the predictive effect of SIIRI in specific populations, such as patients with subclinical coronary atherosclerosis, remains to be investigated.^45^

A study by Li et al.^20^ evaluated the impact of five inflammatory markers on prognosis in patients with ACS and found that SIRI had the strongest predictive power and improved diagnostic efficacy when combined with the GRACE score. Our study combined six novel lymphocyte‐based inflammatory markers with traditional risk factor‐based models to further illustrate their predictive power. We found that the C‐index of all six inflammatory markers was significantly improved when combined with traditional risk factor models. However, only the NRI and IDI values of SIIRI significantly differed from those before the combination. Therefore, SIIRI, serving as a novel marker of inflammatory response, has the potential to serve as an indicator of residual inflammatory risk, akin to high‐sensitivity C‐reactive protein. This could aid in the precise identification of high‐risk individuals with recurrent coronary heart disease. Subsequently, effective management of comorbidities in these patients, including the administration of anti‐inflammatory medications to mitigate the inflammatory response and the implementation of more proactive coronary interventional examinations, may be facilitated. These interventions collectively have the potential to contribute to a reduction in MACE.

This study has certain limitations. First, this study is a small, single‐center, retrospective, observational study, which may have a selection bias in the enrolled population which limit the conclusions that can be made. Furthermore, it is necessary to conduct larger studies to reevaluate the risk assessment of MACEs subtypes, such as specific mortality, recurrent myocardial infarction and stroke, as the current analysis is limited by the small sample size and low incidence of positive outcomes. Second, since our study was conducted only in China, it must be extended to other ethnic groups for further verification. Concurrently, due to the absence of height and weight data for many patients in the primary dataset, this research was unable to incorporate obesity as a prognostic factor. Future investigations should focus on the patients' body mass index to ascertain its predictive significance. Third, our criterion for quantifying the severity of CAD at presentation was the CASSC score, which did not have the power to account for the biased effect of plaque stability. Fourth, our study included a relatively wide range of exclusion criteria, and further studies are needed to explore the predictive value of inflammatory markers in the presence of these exclusion criteria to expand its application to other populations. Fifth, the combination of traditional risk factors and SIIRI has weak diagnostic enhancement efficiency, and SIIRI may need to be combined with other noninflammatory indicators to improve the diagnostic efficiency of the model. Finally, we did not investigate the association between the dynamic changes in inflammatory markers during patient follow‐up and the incidence of MACEs, which needs to be explored in future studies.

## 5 CONCLUSIONS

SIIRI ≥ 247.170 × 10^18^/L^2^ is independently associated with MACEs in newly diagnosed CAD patients. SIIRI was superior to the other five indicators in predicting the occurrence of MACEs. However, combining SIIRI with the traditional risk factors model can better predict MACEs. In future studies, we plan to add SIIRI to traditional risk factor models to complement inflammatory deficits. The optimal cutoff value of SIIRI and its applicable population need to be further explored.

## AUTHOR CONTRIBUTIONS

Li and Liu designed the research study. Li performed the research. Initials Chen provided help and advice on reference search. Gao analyzed the data. Guo and Bai wrote the manuscript. All authors contributed to editorial changes in the manuscript. All authors read and approved the final manuscript.

## CONFLICT OF INTEREST STATEMENT

The authors declare no conflicts of interest.

## ETHICS STATEMENT

The study involving human participants was reviewed and approved by the institutional review Board of the Second Hospital of Tianjin Medical University (IRB number: 2023‐05‐B023). Patients/participants signed written informed consent to participate in this study.

## Data Availability

The data that support the findings of this study are available from the corresponding author, but restrictions apply to the availability of these data, which were used under license for the current study, and so are not publicly available. Data are, however, available from the authors upon reasonable request and with permission of the corresponding author. It can be obtained by contacting the corresponding author when appropriate
